# Eccentric Force-Velocity Characteristics during a Novel Squat Protocol in Trained Rugby Union Athletes—Pilot Study

**DOI:** 10.3390/jfmk6020032

**Published:** 2021-03-30

**Authors:** Conor McNeill, C. Martyn Beaven, Daniel T. McMaster, Nicholas Gill

**Affiliations:** 1 Adams Centre, Te Huataki Waiora School of Health, The University of Waikato, Tauranga 3116, New Zealand; martyn.beaven@waikato.ac.nz (C.M.B.); doctormcmaster@gmail.com (D.T.M.); nicholas.gill@nzrugby.co.nz (N.G.); 2 New Zealand Rugby Union, Wellington 6011, New Zealand

**Keywords:** energy absorption, performance, strength, team sport, kinetic and kinematic

## Abstract

Eccentric strength characteristics have been shown to be important factors in physical performance. Many eccentric tests have been performed in isolation or with supramaximal loading. The purpose of this study was to investigate within- and between- session reliability of an incremental eccentric back squat protocol. Force plates and a linear position transducer captured force-time-displacement data across six loading conditions, separated by at least seven days. The reliability of eccentric specific measurements was assessed using coefficient of variation (CV), change in mean, and intraclass correlation coefficient (ICC). Eccentric peak force demonstrated good ICC (≥0.82) and TE (≤7.3%) for each load. Variables based on mean data were generally less reliable (e.g., mean rate of force development, mean force, mean velocity). This novel protocol meets acceptable levels of reliability for different eccentric-specific measurements although the extent to which these variables affect dynamic performance requires further research.

## 1. Introduction

Eccentric-based training (ECC) has been shown to be an effective strategy for improving physical performance in athletic populations when compared to traditional or concentric-only programs [1,2,3]. The relationship between eccentric phase characteristics and dynamic performance has been previously explored [4,5] and may explain favourable changes in strength, jumping, and sprinting ability following eccentric-based training interventions. Indeed, the stretch-shortening cycle is a well-documented phenomenon in which elastic potential energy stored during the eccentric phase is reutilised to augment the subsequent concentric action [6]. Currently, there is a lack of submaximal eccentric assessments for strength and power development and existing research appears to commonly use concentric strength as a proxy for eccentric-specific exercise prescription [7,8,9,10]. The discrepancy between maximal concentric and eccentric strength has been reported as approximately 20% to 60% depending on the testing procedures [11,12]. Thus, the investigation of a prescription tool for the purpose of eccentric program design is warranted.

The efficacy of ECC methods to improve performance is likely of interest to strength and conditioning practitioners, but there appears to be a lack of standardisation around the practical assessment of eccentric-specific characteristics. Meylan et al. [13] reviewed different protocols for assessing eccentric strength and reported a lack of available reliability statistics and questioned the practicality of existing options. Recently, Bogdanis et al. [14] investigated a submaximal, eccentric-only protocol with university students but only reported relative reliability for the within-session values. Other researchers [5,11] have utilised a three-second eccentric squat to determine maximal eccentric strength in athletes that was largely dependent on subjective determination of the failure threshold. Douglas et al. [15] addressed this by adding an objective velocity standard to their testing protocol. However, the nature of a maximal eccentric test typically relies on extremely heavy loading and forces applied to the musculo-skeleton system, which may limit the applicability in sports as a result of potential muscle damage, soreness, decreased sport performance and recovery time [16].

A recent review has reported on the concentric force-velocity relationship in single fibre, and in vivo investigations suggesting a hyperbolic shape, while the inclusion of the eccentric phase produces a sigmoidal curve around zero velocity [17]. This effect is mirrored in earlier research that suggests the existence of a plateau in force production beyond a certain limit, potentially as a result of neural inhibition, which may diminish with training [18]. The monitoring of force-velocity characteristics during incremental multi-joint assessment has allowed for the accurate estimation of maximum strength in dynamic movement [19]. At present there are limited eccentric-specific testing options available to practitioners, therefore, an incremental, submaximal protocol may provide valuable insight into the characteristics of an individual’s eccentric force-velocity profile.

The aim of this pilot study was to investigate between- and within-session reliability for novel force-velocity data during the eccentric phase of a barbell back squat with participants having the intent of maximizing downward velocity. We hypothesised that following two familiarisation sessions, the results of this experimental protocol with trained athletes would meet commonly applied standards of reliability. In order to make these findings applicable to a practical sport environment, trained athletes were recruited to perform the barbell back squat under standardised incremental loading conditions.

## 2. Materials and Methods

### 2.1. Subjects

Twenty-four semi-professional, male rugby-union athletes were recruited to participate in this study. Age (20.8 ± 2.0 y), height (185.6 ± 6.6 cm), and body mass (100.4 ± 13.7 kg) were recorded prior to the initial testing session. Participants were asked to refrain from strenuous activity at least 24 h prior to testing and to maintain their normal diet on testing days. Inclusion criteria were males 18 years or older, two years of resistance training experience, participation in provincial-level rugby union or higher, and free from any significant musculoskeletal injury or illness occurring within the last month. All participants gave their written consent to participate after being informed through written and oral description of the research project and all relevant information. This research project was approved by the Human Research Ethics Committee of the University of Waikato on 24 October 2018 (HREC[Health]2018#60).

### 2.2. Study Design

**Figure 1:**
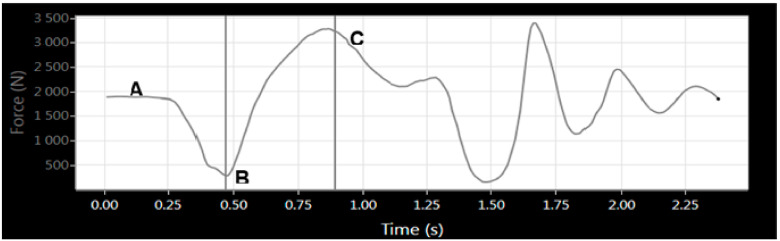
Example of a force trace during an incremental eccentric back squat with a trained rugby union athlete. (**A**) represents the standing position with the barbell held across the trapezius muscles. The vertical line at (**B**) is the point of minimum force and is considered the beginning of the eccentric phase. (**C**) coincides with the point of lowest displacement and the end of the eccentric phase.

Participants completed all familiarisation and testing sessions in their normal training environment at approximately the same time as their normal training sessions to minimise the effects of diurnal variation (between 5 am and 8 am). Further, participants were asked to maintain their normal nutritional practices prior to each testing session. A test–retest reliability design was used with a smaller subset of participants (*n* = 13; age = 21.2 ± 2.2 y; height = 184.9 ± 8.0 cm; body mass = 102.2 ± 15.5 kg) completing a second testing session. The drop out in the second trial was due to scheduling conflicts (*n* = 6) or not showing up to scheduled testing sessions (*n* = 5). At least seven days separated each testing session to allow for recovery from delayed-onset muscle soreness [16]. Kinetic and kinematic data were captured for each repetition performed during the eccentric squat assessment. Specifically, eccentric peak force (EPF), eccentric peak velocity (EPV), eccentric mean force (EMF), eccentric mean velocity (EMV), eccentric mean rate of force development (RFD), range of motion (RoM), and duration of the eccentric phase (duration) were analysed during the first and second testing sessions. The initiation of the eccentric phase was defined as the point of minimum force recorded during the downward phase of movement which was identified manually by the primary investigator [20]. The end of the eccentric phase was determined by the software and was considered to be the lowest point of vertical displacement (Figure 1).

### 2.3. Experimental Procedures

Prior to the initial testing, participants reported to the training facility to complete two familiarisation sessions separated by 24 h. A dynamic, bodyweight warm-up routine was performed consisting of five minutes of stationary biking at a self-selected pace followed by: good mornings, single leg squats (Bulgarian squat), core stability (dead bugs), shoulder internal/external rotation, and hip internal/external rotation for two sets of ten repetitions each (side) and 90–120 s rest between sets. This warm-up procedure was performed before each familiarisation and testing session. Participants were asked to back squat to a self-selected depth approximating 90° at the knee using three standardised loads (wooden dowel/~300 g, 60 kg, and 80 kg) for ten, five, and five repetitions, respectively, with 90–120 s rest between sets. The participants received verbal instructions to perform the first repetition in a “slow and controlled” manner with a “two seconds down, two seconds up” tempo and then progressively increase the eccentric and concentric velocity such that the last repetition was performed at maximal velocity. A digital timer was provided to assist with the tempo of the initial repetitions and the rest period between sets.

Following the dynamic warm-up, each participant completed the novel eccentric force-velocity assessment using six different absolute loads in the barbell back squat (20, 40, 60, 80, 100, and 120 kg). The manner of testing was consistent in both trials, with loads proceeding in ascending order. Participants unable to complete three repetitions at a given load were excused from that load (120 kg; Trial 1, *n* = 5; Trial 2, *n* = 2). Technique was standardised with feet placed shoulder width apart and toes turned slightly out. A barbell was placed across the trapezius with hands placed comfortably on the barbell. Participants were asked to descend until they reached 90° of flexion at the knee. Each attempt was performed on two force plates (PASCO Scientific Inc., Roseville, CA, USA) with a linear position transducer (Celesco Transducer Products, Chatsworth, CA, USA) attached to the barbell just inside the collar, positioned laterally to the participant’s centre of mass. Custom-made software (Weightroom, HPSNZ, Auckland, New Zealand) down sampled the signal to 100 Hz and 250 Hz for ground reaction forces and linear displacement, respectively. The same equipment was used by the same operator in all testing sessions. Previous research has shown this frequency provides reliable ground reaction force data during athletic testing [21].

Participants were asked to perform three dynamic repetitions at each load while attempting to maximise velocity in the eccentric and concentric phases. Each participant was asked to remain motionless between repetitions and was given approximately three to five minutes rest between sets [22]. Loud music was played during each testing session over the gym speaker system, and verbal encouragement was given during each attempt. The movement cues for the assessment were standardised as “fast down, fast up”, “move as quickly as possible”, and “squat to your normal depth. Further, participants were asked to keep their feet in contact with the ground throughout the trial to minimise movement variation. A certified strength and conditioning specialist oversaw all familiarisation and testing sessions to ensure participants understood the procedures.

### 2.4. Statistical Analyses

Data were initially log-transformed for reliability analysis to reduce bias from non-uniformity of error and are presented as mean ± SD or 90% confidence limits. Study data is included as Supplementary Materials. Intraclass correlation coefficient (ICC), coefficient of variation (CV), and the change in mean were calculated for EPF, EPV, EMF, EMV, and RFD using customised Excel spreadsheets [23]. Two-way mixed effects ICCs (3,1) were interpreted accordingly: <0.4 poor, 0.4 to 0.75 fair, 0.75 to 0.9 good, and >0.9 excellent [24,25]. Between-session analysis was comprised of mean values for each trial. Within-session analysis was conducted on the three repetitions completed for each load in Trial 1.

Trials and repetitions were assessed for systematic error (i.e., learning effects) using repeated measures analysis of variance (Rstudio, version 1.2.5033 with R version 3.6.2). Generalised eta squared (η 2 G) effect sizes for repeated measures were interpreted as <0.02 as trivial, 0.02 to 0.13 as small, 0.13 to 0.26 as medium, and >0.26 as large [26]. A Bonferroni-Holm post hoc test was performed if significant differences were found. The alpha level for significance was set at *p* ≤ 0.05. If the assumption of sphericity was violated, the adjusted *p*-values were reported. If systematic error was present the repetition or trial was either excluded or the measurement schedule was modified [27].

## 3. Results

Within-session, one-way repeated measures ANOVA revealed a significant main effect for repetitions in each variable tested (*p* < 0.001 for all variables; EMF, η 2 G = 0.015; EMV, η 2 G = 0.179; EPF, η 2 G = 0.030 EPV, η 2 G = 0.175; Mean RFD, η 2 G = 0.048; Duration, η 2 G = 0.032; RoM, η 2 G = 0.125). Post hoc comparisons revealed that repetition A was significantly different from B and C across all variables (*p* < 0.001). No significant differences were detected between B and C with the exception of eccentric duration (*p* = 0.04). Reliability analysis shows consistently larger changes in the mean and lower absolute reliability (CV) for each variable when repetition A is included (Table 1). Specifically, range of motion and the duration of the eccentric phase tended to have higher absolute reliability when repetition A was excluded. These measures may suggest the presence of systematic error (i.e., learning effect, protective strategies) between repetitions and thus repetition A was excluded from the test–retest analysis [28].

**Figure 2:**
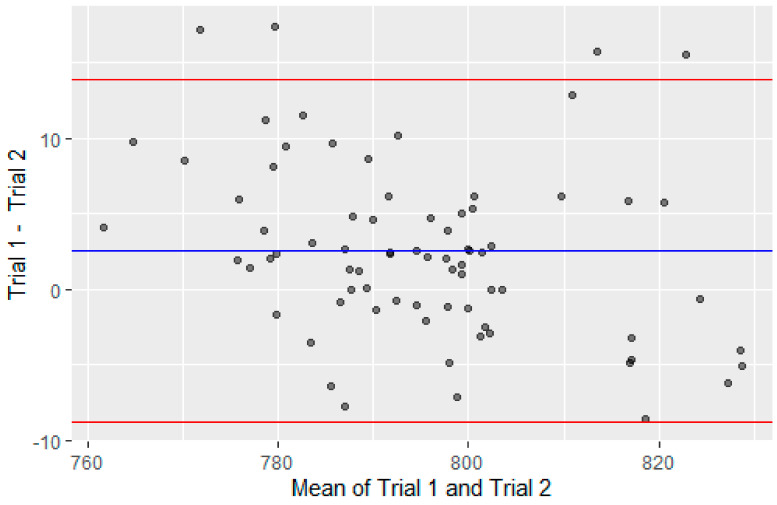
Bland–Altman analysis for between trial differences for eccentric back squat force-velocity assessment in trained athletes. Data shown is for log-transformed eccentric peak force across all loads tested.

No significant differences were found in the between-session ANOVA except for EPF (*p* = 0.049, η 2 G = 0.006) suggesting that participants were adequately familiarised with the testing procedure (EMF, *p* = 0.46, η 2 G = 0.005; EMV, *p* = 0.71, η 2 G = 0.001; EPV, *p* = 0.77, η 2 G = 0.001; Mean RFD, *p* = 0.89, η 2 G < 0.001; Duration, *p* = 0.33, η 2 G = 0.006; RoM, *p* = 0.90, η 2 G < 0.001). The *p*-value for EPF was found to be less than the alpha level for significance, but the effect size was trivial therefore the results for EPF were interpreted as having acceptable between -session reliability. Relative (ICC) and absolute (CV) measures of reliability differed across the variables tested (Table 2). EMF, EMV, and EPV resulted in poor to good relative reliability depending on the load while absolute reliability ranged from 2.4% to 15.5%. EPF demonstrated good relative reliability (≥0.82) and an absolute reliability of ≤7.3% for each load. A Bland–Altman plot for differences between trials in shown in Figure 2. Mean rate of force development tended to show the lowest levels of both relative and absolute reliability when 90% confidence limits were included.

## 4. Discussion

To our knowledge, this is the first study to examine the reliability of aspects of the eccentric phase across different loading conditions performed with the intent to maximise velocity in trained athletes. This investigation utilised a well-accustomed exercise in the participants’ normal training environment. The findings of the current study suggest that analysis of EPF has good reliability across loading conditions. EPV, EMV, and EMF have CV values under the commonly applied threshold of 10%; however, we acknowledge that this value constitutes an arbitrary cut-off point [29]. Based on the specific model of ICC (3,1) in this study, practitioners should be cautious when inferring results to other populations and testing conditions.

The results of this investigation were in agreement with our hypothesis that force and velocity results from an eccentric back squat assessment are reliable following familiarisation of the testing protocols. Hansen et al. [20] investigated different methods of quantifying force-time variables, noting that peak values may be a more reliable measure as these are not dependent on beginning and end points. The mean values found in this investigation are in agreement with Hansen as they tended to have lower reliability, especially in mean rate of force development. Pérez-Castilla et al. [30] also reported smaller CV values during the eccentric phase of mean velocity and mean power during a loaded counter-movement jump. The relative reliability of EPF in our study appears to be similar to eccentric peak force values found by Frohm et al. [31] in their investigation of a supramaximal protocol.

Within-session variability was found to be significantly greater during the first repetition, and as a result, was removed when conducting test–retest analysis. As noted in Table 1, the change in mean after the first repetition is consistently positive in force and velocity variables. One explanation for this might be that participants were using the initial repetition as “practice” or “warm up” which may explain the subsequent improvements in performance. Hopkins et al. [28] noticed that error values were consistently larger between the first two trials but smaller in subsequent trials for studies investigating power. Future testing and study designs should accommodate for this variability between initial and subsequent trials.

The results of the current study demonstrate novel findings with regard to the eccentric characteristics of trained athletes in a familiar exercise. The relationship with load was found to differ between the variables of interest used in the investigation (Table 2). EPF tended to demonstrate a non-linear trend as barbell load increased while EMF increased concomitantly with load. This discrepancy in ground reaction forces (GRF) may have implications in training program design when the goal is to expose athletes to higher barbell loads or greater magnitudes of GRF. In a recent study investigating ECC with academy rugby athletes, the authors found small differences between fast and slow ECC groups both with and without the inclusion of accentuated loading. The eccentric loads used in that study ranged from 74% to 110% of concentric 1RM. By comparison, our study noted a plateau in peak force after 60 kg which may have been notably less than the loads used in their investigation. Therefore, although the loading schemes, and likely mean forces, differed between experimental groups, the groups may have been exposed to similar peak forces. We postulate that the novel findings from our study may help facilitate training load selection in future studies that elicit distinct GRF between experimental groups. Practitioners wanting to implement ECC should consider programming variables such as load, velocity, GRF and their effect on the resultant adaptation to the specific demands imposed by these methods.

### Limitations

The authors acknowledge that maximal strength was not tested and used to prescribe individualised testing loads. Maximal strength likely varied between individuals in the current study and thus each absolute load represented a different percentage of an individual’s ability. The extent to which relative strength levels played a role in the reliability of different measures is unclear. However, EPF reliability was found to be consistent across each of the loads tested. The barbell loads in this investigation were exploratory and represented a wide spectrum of potential force-velocity outcomes, although it is noted that these loads exceeded the strength capabilities of some individuals accounting for the drop out at 120 kg. The manner in which testing proceeded, in an ascending order, was chosen by the authors to allow for data collection in a practical environment. Sufficient rest was provided to minimise any fatigue effect, with rest periods aligning with guidelines for maximal concentric strength testing [22]. Additionally, sampling frequency may have played a role in the results from this investigation. While 100 Hz has been shown to meet minimum standards of reliability, Hori et al. [21] found that reductions in precision were noted below a 200 Hz threshold. Future studies examining reliability in eccentric variables should consider using testing instruments with higher sampling frequencies.

## 5. Conclusions

The goal of this investigation was to determine the reliability of force and velocity values during the eccentric phase of a novel back squat test in trained athletes. Based on these results eccentric peak force has the highest absolute and relative reliability across all loads tested. Future authors wanting to investigate eccentric-specific outcomes may explore force and velocity variables at a range of loading parameters rather than strictly maximal eccentric conditions. The authors postulate the dynamic contractions used in this study may have a stronger relationship to the specific demands of rugby union than traditional physical testing. This protocol provides strength and conditioning professionals with a novel tool for understanding eccentric-specific changes following targeted training interventions. Practitioners wanting to implement ECC based on the relationships observed in this study may adjust loading strategies (heavier or lighter loads) to maximise the desired programming outcomes (GRF, EPV, etc.) Although the generalisation of these findings is limited to the current sample, practitioners may implement this test to determine reliability with their own athletes. Caution should be used when interpreting rate of force development measures as these have shown greater measurement error than those derived from peak values. The contribution of these variables to physical performance as well as the sensitivity to change following longitudinal training interventions warrants further research.

## Figures and Tables

**Table 1:** Reliability and change scores for within-session repetition comparison in an eccentric back squat test.

	CV (%)	Change in Mean (%)
**EPV**		
AB	5.8 (5.3 to 6.5)	12.2 (11.0 to 13.5)
AC	7.0 (6.3 to 7.8)	11.8 (10.3 to 13.3)
BC	5.7 (5.2 to 6.3)	−0.4 (−1.5 to 0.7)
**EPF**		
AB	3.7 (3.3 to 4.1)	6.0 (5.2 to 6.7)
AC	4.5 (4.1 to 5.0)	5.8 (4.9 to 6.7)
BC	3.2 (2.9 to 3.6)	−0.2 (−0.8 to 0.5)
**EMV**		
AB	6.1 (5.5 to 6.8)	12.7 (11.4 to 14.0)
AC	7.6 (6.9 to 8.4)	11.9 (10.2 to 13.5)
BC	6.1 (5.5 to 6.8)	−0.7 (−1.9 to 0.4)
**EMF**		
AB	4.8 (4.3 to 5.3)	3.2 (2.3 to 4.2)
AC	4.6 (4.2 to 5.1)	2.7 (1.8 to 3.6)
BC	4.0 (3.6 to 4.5)	−0.5 (−1.3 to 0.3)
**RFD**		
AB	13.7 (12.4 to 15.4)	15.4 (12.5 to 18.4)
AC	15.6 (14.1 to 17.5)	12.7 (9.5 to 16.0)
BC	13.6 (12.3 to 15.2)	−2.3 (−4.7 to 0.2)
**RoM**		
AB	4.9 (4.3 to 5.8)	7.6 (6.1 to 9.1)
AC	6.6 (5.7 to 7.7)	8.1 (6.1 to 10.2)
BC	2.0 (1.7 to 2.3)	1.7 (1.1 to 2.3)
**Duration**		
AB	6.5 (5.9 to 7.3)	−5.3 (−6.5 to −4.1)
AC	7.4 (6.7 to 8.2)	−4.3 (−5.6 to −2.9)
BC	6.8 (6.2 to 7.6)	2.4 (1.1 to 3.8)

Notes: CV and change in mean values were calculated with log-transformed data. Values are presented with 90% confidence limits. Sample size for 120 kg, *n* = 19; for all other loads *n* = 24. Abbreviations: ABC = first, second, and third repetition of the test, respectively; EMF = eccentric mean force; EMV = eccentric mean velocity; RFD = eccentric mean rate of force development; EPF = eccentric peak force; EPV = eccentric peak velocity; RoM = eccentric range of motion; Duration = time duration of eccentric phase.

**Table 2:** Between-session reliability for eccentric force-time-displacement variables obtained with different loads in the back squat.

	Trial 1 ± SD	Trial 2 ± SD	% Change in Mean (90% CL)	% CV (90% CL)	ICC (90% CL)
**EPF**					
20	2682.7 ± 425.7	2661.7 ± 537.3	−1.4 (−4.3 to 1.5)	4.3 (3.3 to 6.7)	0.95 (0.88 to 0.98) *
40	2818.3 ± 420.5	2808.0 ± 436.5	−0.5 (−3.3 to 2.3)	4.1 (3.1 to 6.3)	0.94 (0.84 to 0.98) *
60	2996.9 ± 503.6	2852.8 ± 462.3	−4.8 (−7.8 to −1.7)	4.7 (3.6 to 7.3)	0.93 (0.83 to 0.97) *
80	3006.9 ± 421.6	2911.9 ± 493.9	−3.5 (−6.5 to −0.5)	4.5 (3.3 to 6.8)	0.93 (0.82 to 0.97) *
100	2994.3 ± 392.3	2925.5 ± 460.3	−2.6 (−5.3 to 0.2)	4.1 (3.1 to 6.3)	0.93 (0.82 to 0.97) *
120	2941.0 ± 385.1	2898.8 ± 459.1	−1.7 (−4.1 to 0.7)	3.2 (2.4 to 5.2)	0.96 (0.88 to 0.99) *
**EPV**					
20	2.04 ± 0.25	2.11 ± 0.26	3.8 (−0.4 to 8.2)	6.1 (4.6 to 9.4)	0.81 (0.56 to 0.92)
40	1.94 ± 0.21	1.98 ± 0.22	2.0 (−1.9 to 5.9)	5.6 (4.2 to 8.6)	0.79 (0.52 to 0.92)
60	1.81 ± 0.19	1.79 ± 0.19	−1.2 (−3.9 to 1.6)	4.0 (3.0 to 6.1)	0.88 (0.71 to 0.95)
80	1.59 ± 0.20	1.61 ± 0.18	1.5 (−3.0 to 6.1)	6.6 (4.9 to 10.1)	0.74 (0.42 to 0.89)
100	1.40 ± 0.18	1.41 ± 0.16	0.9 (−2.7 to 4.7)	5.4 (4.1 to 8.3)	0.84 (0.61 to 0.94)
120	1.24 ± 0.22	1.25 ± 0.22	0.9 (−5.9 to 8.2)	9.5 (6.9 to 15.5)	0.80 (0.49 to 0.93)
**EMF**					
20	1453.5 ± 213.1	1452.8 ± 267.9	−0.5 (−4.2 to 3.3)	5.5 (4.1 to 8.5)	0.91 (0.78 to 0.97) *
40	1611.7 ± 158.4	1573.0 ± 232.3	−2.9 (−7.9 to 2.3)	7.8 (5.9 to 12.1)	0.65 (0.28 to 0.85)
60	1835.3 ± 176.7	1807.4 ± 208.3	−1.7 (−5.4 to 2.2)	5.7 (4.3 to 8.7)	0.77 (0.48 to 0.91)
80	2015.6 ± 167.5	1949.4 ± 188.9	−3.4 (−6.6 to 0.0)	5.0 (3.7 to 7.6)	0.74 (0.43 to 0.90)
100	2187.7 ± 175.2	2121.0 ± 197.7	−3.2 (−6.3 to 0.1)	4.8 (3.6 to 7.3)	0.75 (0.44 to 0.90)
120	2273.7 ± 126.7	2229.1 ± 180.3	−2.1 (−4.9 to 0.7)	3.8 (2.8 to 6.1)	0.77 (0.43 to 0.91)
**EMV**					
20	1.43 ± 0.15	1.48 ± 0.18	3.3 (−0.5 to 7.3)	5.5 (4.1 to 8.4)	0.80 (0.55 to 0.92)
40	1.33 ± 0.11	1.35 ± 0.14	1.1 (−2.7 to 5.0)	5.7 (4.2 to 8.7)	0.73 (0.41 to 0.89)
60	1.23 ± 0.14	1.21 ± 0.14	−1.4 (−4.0 to 1.3)	3.9 (2.9 to 6.0)	0.91 (0.76 to 0.96) *
80	1.08 ± 0.13	1.07 ± 0.13	−0.1 (−3.8 to 3.7)	5.6 (4.2 to 8.5)	0.83 (0.60 to 0.93)
100	0.92 ± 0.14	0.92 ± 0.13	−0.3 (−4.6 to 4.1)	6.5 (4.9 to 10.0)	0.82 (0.59 to 0.93)
120	0.79 ± 0.17	0.78 ± 0.18	−1.9 (−7.5 to 4.1)	7.9 (5.8 to 13.0)	0.92 (0.77 to 0.97) *
**RFD**					
20	6631.6 ± 1682.1	7144.9 ± 2309.0	6.1 (−1.3 to 14.0)	10.9 (8.1 to 16.9)	0.90 (0.75 to 0.96)
40	6266.1 ± 1286.9	6425.4 ± 1427.9	2.4 (−4.9 to 10.3)	11.2 (8.3 to 17.4)	0.81 (0.56 to 0.92)
60	6053.9 ± 1494.8	5781.8 ± 1370.4	−4.3 (−9.8 to 1.5)	8.9 (6.6 to 13.8)	0.90 (0.76 to 0.96) *
80	5101.4 ± 1296.3	4979.0 ± 1306.8	−2.8 (−11.8 to 7.2)	15.0 (11.1 to 23.6)	0.74 (0.42 to 0.89)
100	4120.8 ± 1114.0	4125.1 ± 1084.2	0.6 (−12.8 to 16.0)	22.7 (16.7 to 36.3)	0.50 (0.05 to 0.78)
120	3388.4 ± 1180.5	3353.3 ± 1364.8	−2.1 (−16.3 to 14.5)	22.5 (16.2 to 38.1)	0.81 (0.51 to 0.93)

Notes: Mean values are presented as raw data, while coefficient of variation (CV) and intraclass correlation coefficient (ICC) values were calculated with log-transformed data. Values are presented with ± standard deviation (SD) or 90% confidence limits (90% CL). Asterisk (*) denotes an ICC above 0.75 (based on confidence limits). Sample size for 120 kg, *n* = 11; for all other loads *n* = 13. Abbreviations: ABC = first, second, and third repetition of the test, respectively; EMF = eccentric mean force; EMV = eccentric mean velocity; RFD = eccentric mean rate of force development; EPF = eccentric peak force; EPV = eccentric peak velocity.

## Data Availability

The data presented in this study are available as Supplementary Materials.

## Supplementary Materials

The following are available online at https://www.mdpi.com/article/10.3390/jfmk6020032/s1.
